# Haematological reference intervals for healthy adults in Bamenda, Cameroon

**DOI:** 10.4102/ajlm.v9i1.1193

**Published:** 2020-12-21

**Authors:** Victor N. Fondoh, Richard M. Fondoh, Charles N. Awasom, Pefoule L. Edith, Winlove A. Ntungwen, Bong Roland, Rebeca Enow-Tanjong, Patrick Njukeng, Judith Shang, Egbe P. Egbengu, Talkmore Maruta, Akazong Etheline, Robert Leke, Ayuk Leo, Denis Nsame

**Affiliations:** 1 Administration/Quality Management, Bamenda Regional Hospital Laboratory, Regional Hospital Bamenda, Bamenda, Cameroon; 2 Department of Medical Laboratory Sciences, School of Health and Medical Sciences, Catholic University of Cameroon, Bamenda, Cameroon; 3 Department of Health Economics Policy and Management, Faculty of Business Management, University of Cameroon, Bamenda, Cameroon; 4 Administration/Pharmaceutical Management, North-West Regional Fund for Health Promotion, Bamenda, Cameroon; 5 Department of Anatomy, School of Health and Medical Sciences, Catholic University of Cameroon, Bamenda, Cameroon; 6 Bamenda Regional Hospital Laboratory, Regional Hospital Bamenda, Cameroon; 7 Patient First Laboratory, Columbia, Maryland, United States; 8 Product Safety/Quality Control Mangement, Geochim Sarl, Cameroon; 9 Department of Medical Laboratory Science, School of Health and Medical Sciences , Catholic University of Cameroon, Bamenda, Cameroon; 10 Global Health Systems Solutions, Limbe, Cameroon; 11 Laboratory Service, Center for Disease Control and Prevention, Yaoundé, Cameroon; 12 Department of Medicine and Surgery, School of Health and Medical Sciences, Catholic University of Cameroon, Bamenda, Cameroon; 13 East Central and Southern Africa Health Community, Arusha, United Republic of Tanzania; 14 Department of Biochemistry, University of Dschang, Dschang, Cameroon; 15 Department of Medicine and Surgery, School of Health and Medical Sciences, Catholic University of Cameroon, Bamenda, Cameroon; 16 TB-Department, Regional Hospital Bamenda, Bamenda, Cameroon; 17 Administration/Management, Regional Hospital Bemenda, Bamenda, Cameroon

**Keywords:** haematological reference intervals, African population, pathogenic infections, haematological abnormalities, Cameroon, Clinical and Laboratory Standard Institute, local reference values, Bamenda

## Abstract

**Background:**

In the era of evidence-based medicine, haematological reference intervals are essential for the interpretation of data for clinical decision-making, monitoring of treatment and research. It is not uncommon that reference intervals used in most African countries have been obtained from published scientific literature, textbooks, reagent/instrument manuals.

**Objective:**

The aim of this study was to determine haematological reference intervals of healthy adults in Bamenda, Cameroon.

**Methods:**

This was a cross-sectional study conducted between June and November 2015. Participants were voluntary blood donors at the Blood Bank Service of the Regional Hospital Bamenda aged between 18 and 65 years. The mean, median and standard deviation of the mean were calculated for each haematological parameter. The 95th percentile reference intervals were determined using the 2.5th and 97.5th percentile. The differences between gender for all the parameters were evaluated using the Kruskal-Wallis test. Significance was determined at the 95% confidence level.

**Results:**

Out of a total of 340 participants, 202 (59.4%) were men and 138 (40.6%) were women. The median red blood cell, haemoglobin, haematocrit and mean cell haemoglobin concentration were significantly higher in men than women (*p* < 0.001). The median white blood cell, absolute lymphocytes count, absolute granulocytes and platelet counts for men were significantly lower than those for women (*p* < 0.011).

**Conclusion:**

We propose that the present established haematological reference intervals in this study should be used for clinical management of patients and interpretation of laboratory data for research in Bamenda.

## Introduction

Haematological reference intervals are essential for the interpretation of data for diagnosis, clinical decision-making and research in this era of evidence-based medicine. It is not uncommon that reference intervals used in most African countries have been obtained from published scientific literature, textbooks,^1,2^ the world wide web, reagent package inserts or instrument manuals.^3^ More often than not, these values have been established from ‘Caucasian’ populations in Europe or the United States and may not apply to local settings.^4,5^ There is published literature to confirm that haematological reference intervals established in African populations^6,7,8,9,10,11,12,13^ differ significantly from those obtained from Caucasian populations.^5,14,15^ Several factors, including inter- and intra-population variation among populations of the same race, age, sex, geographical origin, altitude, genetics, dietary patterns and ethnicity,^7,16,17,18,19,20^ account for the differences in these reference intervals. Moreover, pathogenic infections such as HIV, Hepatitis B Virus (HBV), Hepatitis C Virus (HCV), syphilis and some haematological abnormalities generally influence the haematological intervals.^9,21,22^ Besides, the Clinical and Laboratory Standard Institute recommends that clinical laboratories establish and/or verify their local reference values.^21,23^

Cameroon is one of the countries that has been burdened by the malaria and HIV epidemics and that has received multilevel interventions, including access to drugs, and capacity building to manage prevention, treatment and clinical trials. There is little published literature on haematological reference intervals established for the population of Yaoundé in Cameroon.^6^ These intervals cannot be used nationwide since Yaoundé is not representative of the average topography or ecological niche of Cameroon, in general, and Bamenda, in particular. Besides the fact that Bamenda is at a lower altitude than Yaoundé City and differs from other settlements and ethnic groups, there is a need for clinical laboratories to establish and harmonise standard intervals in all localities^23^ for effective clinical decision-making, monitoring of treatment and management of interventions.^24,25,26,27^ The objective of this study was to determine the haematological reference intervals of healthy adults between April and September 2015 in Bamenda, Cameroon.

## Methods

### Ethical considerations

Ethical clearance to carry out this research was obtained from the Institutional Review Board of Regional Hospital Bamenda, Cameroon (Number: 029/APP/RDPH/RHB/IRB). Participants consented to participate in the study by signing the consent form. Participants could withdraw from the study even after signing the consent form.

### Study area

The study was conducted at the Regional Hospital Bamenda situated in Bamenda, capital of the North-West region of Cameroon, which lies at an altitude between 1100 m and 1430 m above sea level.^28,29^ Because of its high socio-economic activity, Bamenda is a cosmopolitan city with settlements of people from diverse ethic backgrounds,^30^ including Mankon, Nkwen, Bamendakwe, Nsongwa, Mbatu, Chomba and Bandza.^31^ As it is situated in the grass fields, most of their diet includes varieties of vegetables.^32^

Regional Hospital Bamenda has standard clinical laboratory and Blood Bank services. The laboratory has been implementing laboratory quality management systems since 2010 and obtained ISO 15189 accreditation by the South African National Accreditation Services in 2017 for Biochemistry, Serology and Haematology services.^33^ Currently, the Blood Bank service is in the process of certification with the Safe Blood for Africa Foundation.

### Research design

This was a cross-sectional descriptive study conducted between April 2015 and September 2015. The participants were voluntary blood donors who presented during the Regional Hospital Bamenda’s voluntary blood donation programme. Blood samples were collected from the Mankon, Nkwen and Bamendakwe settlements. A stratified and clustered sampling method was used. The population was divided into two groups (men and women) and at least 50 samples were collected from participants at each site and from each sex. The blood donors were subjected to several physical and medical screening protocols, as required by the national blood transfusion programme of the Ministry of Health, Cameroon,^34^ in addition to the Clinical and Laboratory Standards Institute guidelines for the establishment of reference intervals^21^ using a questionnaire.

The questionnaire was used to profile eligible donor. Criteria include: the donor should be free from any non-communicable disease, should not have donated blood or had any sexually-transmitted diseases in the previous three months, should not have been sick or been vaccinated during the previous four months and should not have been on any medication for at least a week before sample collection. Also, the donor should not have smoked on the day of donation or should not have drunk alcohol for at least 24 hours before donation. Female donors should not be pregnant, breastfeeding, or on or expecting their menses within one week of the donation. Furthermore, the donor should be between the ages of 18 and 60 years (women) and 18 and 65 years (men), with blood pressure of 100 mmHg – 140 mmHg/60 mmHg – 100 mmHg, weight greater than 50 kg, and temperature between 36.0 °C – 37.5 °C. Blood specimens were collected from donors who were physically fit and who consented to be part of the study. We anticipated enrolling at least 150 participants from each sex to meet the minimum target of at least 120 or more participants after exclusions, as recommended by the Clinical and Laboratory Standards Institute.^21^

#### Inclusion and exclusion criteria

Participants that met the inclusion criteria for voluntary blood donation were excluded if they were positive for HIV, HBV, HCV or syphilis. Participants who were sickle cell disease carriers (had the AS genotype) or who had sickle cell disease (had the SS genotype) were also excluded. Participants who did not meet the inclusion criteria, who did not consent, or who withdrew their consent after consenting, were excluded.

### Sample collection

Blood was collected by trained and competent personnel into two 5 mL vacutainer tubes containing dipotassium ethylene diamine tetraacetic acid (K_2_EDTA). Samples were stored and transported to the Blood Bank service of the Regional Hospital Bamenda in a cold chain between 2 °C and 8 °C within 2 h of collection. One tube was used for screening HIV, HBV, HCV, syphilis and haemoglobin electrophoresis, and the other for complete blood count analysis. The plasma was separated from the red blood cells in separate tubes within 1 h of the samples’ arrival at the Blood Bank. Both tubes were stored at 4 °C – 8 °C for testing the following day.

### HIV, hepatitis B virus, hepatitis C virus, syphilis and haemoglobin electrophoresis testing

Plasma samples were screened at the Blood Bank department of the Regional Hospital Bamenda. The national algorithm of a rapid test for HIV screening in Cameroon was used.^35^ Samples were screened for HIV using the HIV-1/2 Ag/Ab Combo Determine (Alere Medical Co., Ltd, Matsuhidai, Matsudo-Shi, Chiba-ken, Japan) as the first-line test and OraQuick (OraSure Technologies, Inc., Bethlehem, Pennsylvania, United States) as the second-line test. All participants who were HIV-negative with the first-line test were confirmed as negative with the second-line test. Participants who were positive for HIV with the first-line test only were declared positive and excluded. Syphilis was screened using the Rapid Plasma Reagin carbon slide agglutination assay (Cypress Diagnostics, Langdorp, Belgium) and the *Treponema pallidum* haemagglutination test for the serodiagnosis of syphilis – IMMUTREP^®^ TPHA (Omega Diagnostics LTD, Alva, Scotland, United Kingdom). Hepatitis B virus was screened for using the HBsAg DiaSpot rapid diagnostic test (DIASpot Diagnostics, Jawa Barat, Indonesia) while Hepatitis C virus antigen was detected using the HCV Ag DiaSpot rapid diagnostic test (DIASpot Diagnostics, Jawa Barat, Indonesia). Haemoglobin electrophoresis was done using the Hospitex Diagnostics (Hospitex Diagnostics Srl, Sesto Fiorentino, Italy) electrophoresis machine.

The haematological analysis was done within 6 h of sample collection, using the Urit 3300 auto-analyser (Urit Medical Electronic [Group] Co., Ltd, Guilin, China). The instrument was calibrated using Eurocell Diagnostics internal controls (Eurocell Diagnostics, Rennes Cedex, France), following the protocol provided by the manufacturer. The analyser automatically counted and gave a print-out of results for: red blood cells (RBC); haemoglobin (g/dL); haematocrit (%); mean cell volume; mean cell haemoglobin; mean cell haemoglobin concentration); coefficient of variation for the standard deviation of red cell distribution (%); standard deviation of red cell distribution; white blood cells (WBC); proportion of lymphocytes (%), monocytes (%) and granulocytes (%); absolute count of lymphocytes (×10^9^/L), monocytes (×10^9^/L) and granulocytes (×10^9^/L); platelets; mean platelet volume; platelet distribution width and plateletcrit.

### Quality control

The Urit 3300 auto-analyser used for the analysis of the specimens went through a vigorous formal verification process following the Clinical Laboratory Standards Institute guidelines^21^ and the policies of the quality management system of the Bamenda Regional Hospital Laboratory. Precision was monitored daily using commercial internal controls (Eurocell Diagnostics, Rennes Cedex, France) and reviewed using a Levey-Jennings control chart. Randox International Quality Assurance Scheme RIQAS (Randox Laboratories Limited, Crumlin, County Antrim, United Kingdom) external quality controls were done bi-monthly to monitor accuracy. The analysis was suspended if the daily commercial internal control failed. The analysis was done by a trained and competent technician.

### Data collection

Data were collected by three trained personnel using a structured data collection format. Data for age, sex and haematological parameters for the participants who were negative for HIV, HBV, HCV and syphilis, with no haemoglobin abnormalities, were collected from the print-out of the Urit 3300 auto-analyser. Data were entered into Excel 2007 software (Microsoft Corp., Redmond, Washington, United States) and double-checked for data entry errors by a second person.

### Statistical analysis

The analysis was done using Microsoft Excel 2007 spreadsheet (Microsoft Corporation, Redmond, Washington, United States) and SPSS version 16 software (IBM Corp., Chicago, Illinois, United States). Outliers were eliminated using the box plot function. The median, mean and standard deviation were calculated for each haematological parameter. The 95th percentile reference intervals were determined using the 2.5th and 97.5th percentile. The differences between sexes for all the parameters were evaluated using the Kruskal-Wallis test. Significance was determined at the 95% confidence level.

## Results

**FIGURE 1:**
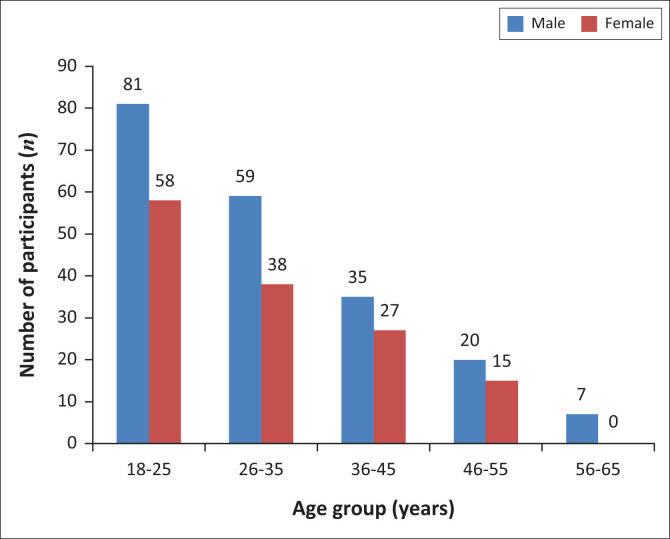
Percentage distribution of age group (years) and sex of participants in the blood donor population of Bamenda, Cameroon, April-September 2015.

Of the 487 individuals who presented for the blood donation campaigns, 147 were excluded as per the exclusion criteria (Table 1). Of the 340 participants included in the study, 202 were male (59.4%) and 138 were female (40.6%) within the age range of 18–60 years (95% confidence interval: 31.5 ± 10.9; median age = 29 years). One hundred and thirty-nine participants (40.9%) were aged 18–25 years, 97 (28.5%) were aged 26–35 years, 62 (18.2%) were aged 36–45 years, 35 (10.3%) were aged 46–55 years, and 7 (2.1%) were aged 56–65 years (Figure 1).

**TABLE 1:** Exclusion criteria applied to the blood donor population, Bamenda, Cameroon, April-September 2015.

Exclusion criteria	Male *n* = 299	Female *n* = 188	Total *N* = 487
Rejected from enrolment as blood donors following the questionnaire	15	8	23
HIV	3	1	4
HBV†	18	6	24
Syphilis†	1	3	4
HCV	4	2	6
AS genotype	51	26	77
Outliers	7	4	11
Total exclusions‡	98	50	148
Population included in the study after exclusion criteria were applied††	202	138	340

HIV, Human Immunodeficiency Virus; HBV, Hepatitis B virus; HCV, Hepatitis C virus; AS, Haemoglobin A and S.

† , One donor was positive for both HBV and syphilis.

‡ , Number of exclusions based on exclusion criteria.

†† , Number of participants who were finally enrolled in the study.

The median RBC, haemoglobin, haematocrit and mean cell haemoglobin concentration were significantly higher in men than in women (RBC: 5.31 × 10^12^/L vs. 4.60 × 10^12^/L, *p* < 0.001; haemoglobin: 14.6 g/dL vs. 12.6 g/dL, *p* < 0.001; haematocrit: 43.9% vs. 38.3%, *p* < 0.001; and mean cell haemoglobin concentration: 33.1 g/dL vs. 32.8 g/dL, *p* = 0.005). Although the median mean cell volume and mean cell haemoglobin were higher in men than women (mean cell volume 27.6 fL vs. 27.3 fL, *p* = 1.000; and mean cell haemoglobin 27.6 pg vs. 27.3 pg, *p* = 0.147), the differences were not statistically significant (Table 2).

**TABLE 2:** Erythrocyte parameter reference intervals of healthy adults stratified by sex, Bamenda, Cameroon, April-September 2015.

Parameters	Red blood cell (×10^12^/L)	Haemoglobin (g/dL)	Haematocrit (%)	MCV (fL)	MCH (pg)	MCHC (g/dL)	RDW_CV (%)	RDW-SD (fL)
**Combined male and female participants (*N* = 340)**								
Median	5.00	13.8	41.8	84.3	27.5	32.9	12.2	45.0
Mean ± SD	5.06 ± 0.71	13.8 ± 1.4	41.7 ± 4.1	83.3 ± 5.9	27.3 ± 2.0	33.1 ± 2.9	12.2 ± 1.2	44.3 ± 5.4
95th percentile interval	4.20–6.11	11.3–16.3	34.3–49.3	69.5–93.2	22.6–31.1	31.6–34.6	10.2–14.9	35.5–52.1
**Men (*N* = 202)**								
Median	5.31	14.6	43.9	84.3	27.6	33.1	12.2	45.0
Mean ± SD	5.30 ± 0.44	14.5 ± 1.1	43.9 ± 3.2	83.3 ± 6.0	27.5 ± 2.1	33.3 ± 3.6	12.1 ± 1.1	44.6 ± 5.4
95th percentile interval	4.42–6.13	12.4–16.4	37.0–49.8	68.2–93.3	22.4–31.6	31.8–34.6	10.2–14.6	35.5–52.1
**Women (*N* = 138)**								
Median	4.60	12.6	38.3	84.3	27.3	32.8	12.2	45.0
Mean ± SD	4.71 ± 0.88	12.6 ± 0.9	38.4 ± 2.8	83.2 ± 5.6	27.2 ± 2.0	32.7 ± 1.2	12.2 ± 1.2	43.8 ± 5.3
95th percentile interval	4.12–5.48	10.9–14.5	32.8–44.2	71.6–92.7	23.1–30.5	31.2–34.4	10.3–15.0	35.5–52.1
*p*-value	< 0.001*	< 0.001*	< 0.001*	1.000	0.147	0.005*	0.89	0.734

RBC, red blood cell; HGB, haemoglobin; HCt, Haematocrit; MCV, mean cell volume; MCH, mean cell haemoglobin; MCHC, mean cell haemoglobin concentration; RDW_CV, coefficient of variation of red cell distribution; RDW-SD, standard deviation of red cell distribution width; *N*, number.

* , *p*-values < 0.05 statistically significant difference between men and women.

The median total WBC, absolute lymphocyte count and absolute granulocyte count were significantly lower in men than in women (WBC: 5.0 × 10^9^/L vs. 5.5 × 10^9^/L, *p* = 0.002; absolute lymphocyte count: 2.1 × 10^9^/L vs. 2.2 × 10^9^/L, *p* = 0.011; and absolute granulocyte count: 2.4 × 10^9^/L vs. 2.8 × 10^9^/L, *p* = 0.002). Although the absolute median monocyte count for men was relatively higher than that of women, the difference was not statistically significant (Table 3).

**TABLE 3:** Leucocyte parameter reference intervals of healthy adults stratified by sex, Bamenda, Cameroon, April-September 2015.

Parameters	White blood cell (×10^9^/L)	Lymphocytes (%)	Monocytes (%)	Granulocytes (%)	Absolute†		
					Lymphocytes (×10^9^/L)	Monocytes (×10^9^/L)	Granulocytes (×10^9^/L)
**Combined male and female participants (*N* = 340)**							
Median	5.3	41.6	8.6	49.4	2.1	0.4	2.6
Mean ± SD	5.4 ± 1.4	42.1 ± 7.4	8.6 ± 1.4	49.2 ± 7.8	2.3 ± 0.7	0.5 ± 0.1	2.7 ± 0.8
95th percentile interval	3.2–8.3	29.2–57.9	6.0–11.4	33.3–63.4	1.3–4.0	0.3–0.8	1.3–4.6
**Men (*N* = 202)**							
Median	5.0	41.8	8.6	49.2	2.1	0.4	2.4
Mean ± SD	5.2 ± 1.3	42.1 ± 7.9	8.6 ± 1.4	49.1 ± 8.2	2.2 ± 0.7	0.5 ± 0.1	2.5 ± 0.8
95th percentile interval	3.0–8.2	28.2–58.0	6.0–11.4	33.1–64.8	1.2–3.8	0.2–0.8	1.3–4.5
**Women (*N* = 138)**							
Median	5.5	41.6	8.4	50.1	2.2	0.5	2.8
Mean ± SD	5.8 ± 1.3	42.0 ± 6.7	8.5 ± 1.5	49.3 ± 7.2	2.4 ± 0.7	0.5 ± 0.2	2.9 ± 0.8
95th percentile interval	3.6–8.3	30.6–56.9	6.0–11.4	34.0–62.4	1.5–4.1	0.3–0.9	1.5–4.7
*p*-value	0.002*	0.896	0.363	0.659	0.011*	0.155	0.002*

SD, standard deviation; WBC, white blood cell; *N*, number.

* , *p*-values < 0.05 statistically significant difference between men and women.

† , number.

Also, the median platelet count was significantly lower in men (231 × 10^9^/L) than in women (253 × 10^9^/L; *p* = 0.009). There was no statistically significant difference between the median mean platelet volume, platelet distribution width and plateletcrit for men as compared to women (Table 4).

**TABLE 4:** Platelet parameter reference intervals of healthy adults stratified by sex, Bamenda, Cameroon, April-September 2015.

Parameters	Platelet (×10^9^/L)	MPV (fL)	PDW (fL)	PCT (%)
**Combined male and female participants (*N* = 340)**				
Median	241	10.0	11.1	0.24
Mean ± SD	243 ± 57.0	10.4 ± 1.6	11.4 ± 2.2	0.25 ± 0.07
95th percentile interval	142.0–354	7.9–13.4	7.9–15.3	0.14–0.41
**Men (*N* = 202)**				
Median	231	10.2	11.1	0.24
Mean ± SD	235 ± 58	10.6 ± 1.7	11.5 ± 2.0	0.25 ± 0.07
95th percentile interval	140–346	8.0–13.4	7.9–15.4	0.11–0.42
**Women (*N* = 138)**				
Median	253	9.9	10.8	0.24
Mean ± SD	253 ± 54	10.2 ± 1.5	11.2 ± 2.3	0.25 ± 0.06
95th percentile interval	148–367	7.8–13.1	7.7–14.9	0.15–0.39
*p*-value	0.009*	0.134	0.633	0.793

MPV, mean platelet volume; PDW, platelet distribution width; PCT, Plateletcrit; SD, standard deviation; *N*, number.

* , *p*-values < 0.05 statistically significant difference between men and women.

## Discussion

The reference interval for haematological parameters, which may serve as a standard for decision-making on clinical laboratory results, treatments and research, were established from 340 participants from Bamenda City, Cameroon. The participants included 202 (59.4%) men and 138 (40.6%) women aged between 18 and 60 years.

According to our findings, the median RBC, haemoglobin and haematocrit for men were significantly higher than for women. These variations may be attributed to the influence of the hormone androgen on erythropoiesis as well as menstrual blood loss in women.^36^ Our findings are consistent with previous reports in Africa, including Oloume et al. in Cameroon,^6^ Awad et al. in Sudan,^38^ Addai-Mensah et al. in Ghana,^7^ Bakrim et al. in Morocco,^8^ Mulu et al. in Ethiopia^13^ and Yalew et al. in Ethiopia,^12^ Miri-Dashe et al. in Nigeria,^36^ Dosoo et al. in Ghana^10^ and Kueviakoe et al. in Togo,^9^ Karita et al. in Eastern and Southern South Africa^21^ and Menard et al. in Central Africa.^20^ Similar findings have also been reported in the United States.^5^ According to Oloume et al. in the Yaounde study in Cameroon, RBC, haemoglobin, haematocrit and mean cell haemoglobin concentration were lower compared to those obtained in this study.^6^ In their study, however, haemoglobin abnormalities were not excluded from their sample collection, considering the 2% prevalence of sickle cell disease in Cameroon^37^ and may thus account for the low values reported. Besides, Bamenda is at a higher altitude than Yaoundé^29,39,40^ and is situated in the grassland; also, its inhabitants are used to the consumption of vegetables^32^ which have a high iron content that may increase the erythrocyte parameters. At higher altitude, there are physiological changes to humans that compensate for the lower partial pressure of oxygen at higher altitudes.^41,42^ The same reason may account for the relatively lower intervals of RBC, haemoglobin and haematocrit in other countries at lower altitudes compared to those in this study,^7,10,12,17,36^ in contrast to higher intervals observed in a study conducted in Ethiopia at higher altitudes.^13,43^ On the other hand, we observed relatively lower intervals of haemoglobin and haematocrit in this study compared to those reported in the United States (Table 5).^5^ This may be attributed to lower ferritin and transferrin saturation among Black participants.^44^ Besides, our study showed a significantly higher mean cell haemoglobin concentration in men than in women, which had also been reported in previous work in Ethiopia.^12^

**TABLE 5:** Comparison of reference intervals in Bamenda, Cameroon, with other African countries and the United States, 2002 and 2015.

Parameters	Gender	Our study Cameroon-Bamenda		Cameroon (Yaoundé)^6^		Eastern and southern South Africa^21^	Central Africa^20^		Ethiopia^13^		Nigeria^36^		Ghana^7^		US-based comparison interval^5^
		95% PI	Median	95% PI	Median	95% PI (Median)	95% PI	Median	95% PI	Median	95% PI	Median	95% PI	Median	95% PI (Median)
RBC (10^12^/L)	Male	4.42–6.13	5.31	4.00–5.90	4.87	4.00–6.40	4.50–6.10	5.14	3.90–6.20	5.34	5.10–5.30	5.20	3.61–6.97	5.19	4.50–5.90
	Female	4.12–5.48	4.60	3.40–5.30	4.11	3.80–5.60	3.42–5.44	4.50	3.90–5.80	4.70	4.50–5.30	4.56	3.08–5.88	4.38	4.00–5.20
Haemoglobin (g/dL)	Male	12.4–16.4	14.6	11.0–16.0	13.5	12.2–17.7	12.3–17.3	14.9	13.6–19.8	16.6	14.0–14.4	14.3	10.7–18.8	15.2	13.5–17.5
	Female	10.9–14.5	12.6	9.9–13.7	11.4	9.5–15.8	9.1–14.9	12.5	12.0–18.0	14.9	12.4–13.1	12.8	8.2–16.2	12.5	12.0–16.0
Haematocrit (%)	Male	37.0–49.8	43.9	34.6–47.6	41.8	35.0–50.8	39–52.0	45.0	45.0–59.0	52.0	43.5–45.0	44.3	31.8–61.8	45.2	41.0–53.0
	Female	32.8–44.2	38.3	29.7–42.0	35.6	29.4–45.4	28.0–44.0	38.0	39.0–55.0	46.1	38.8–40.5	39.7	26.8–50.4	37.4	36.0–46.0
MCV (fL)	Male	68.2–93.3	84.3	70.0–97.0	86.0	NA	NA	-	90.0–109.0	99.0	84.3–86.6	85.8	69.7–103.2	87.1	NA
	Female	71.6–92.7	84.3	72.0–96.0	87.0	NA	NA	-	90.3–106.0	98.7	84.8–86.5	86.1	64.4–103.5	86.8	NA
MCH (pg)	Male	22.4–31.6	27.6	22.8–33.4	28.2	NA	NA	-	28–34.4	31.0	27.2–28.1	27.9	23.3–34.2	29.4	NA
	Female	23.1–30.5	27.3	23.2–31.2	28.1	NA	NA	-	28–34	31.0	27.1–28.9	27.4	19.5–33.7	28.7	NA
MCHC (g/dL)	Male	31.8–34.6	33.1	29.9–35.3	32.3	NA	NA	-	30.0–33.3	32.0	31.9–32.4	32.4	29.7–37.2	33.7	NA
	Female	31.2–34.4	32.8	30.0–34.2	32.1	NA	NA	-	30–33	31.5	31.8–32.3	32.1	26.8–37.1	33.1	NA
RDW (fL)	Male	10.2–14.6	12.2	NA	-	NA	NA	-	NA	-	NA	-	11.7–18.7	14.0	NA
	Female	10.3–15.0	12.2	NA	-	NA	NA	-	NA	-	NA	-	11.8–26.4	14.3	NA
WBC (10^9^/L)	Combined	3.2–8.3	5.3	NA	-	3.1–9.1	NA	-	NA	-	NA	-	NA	-	4.5–11.0
	Male	3.0–8.2	5.0	2.6–6.8	4.1	NA	2.9–8.3	5.1	3.5–10.9	6.7	4.3–4.6	4.4	3.2-11.2	5.5	NA
	Female	3.6–8.3	5.5	2.8–6.7	4.6	NA	2.7–8.0	4.9	3.6–11.9	6.6	4.4–4.8	4.5	3.3–10.6	5.6	NA
Granulocytes (%)	Male	33.1–64.8	49.2	NA	-	NA	NA	-	NA	-	NA	-	NA	-	NA
	Female	34.0–62.4	50.1	NA	-	NA	NA	-	NA	-	NA	-	NA	-	NA
Granulocytes (10^9^)	Male	1.3–4.5	2.4	NA	-	NA	NA	-	NA	-	NA	-	NA	-	NA
	Female	1.5–4.7	2.8	NA	-	NA	NA	-	NA	-	NA	-	NA	-	NA
Lymphocytes (%)	Male	28.2–58.0	41.8	NA	-	NA	NA	-	15.1–53.4	31.1	37.4–40.2	39.0	12.0–66.9	45.7	NA
	Female	30.6–56.9	41.6	NA	-	NA	NA	-	18.0–54.0	32.0	39.0–42.1	40.3	14.6–62.3	41.3	NA
Lymphocytes (10^9^/L)	Male	1.2–3.8	2.1	1.0–3.1	1.7	NA	NA	-	NA	-	NA	-	0.8–4.8	2.4	NA
	Female	1.5–4.1	2.2	1.1–2.9	1.9	NA	NA	-	NA	-	NA	-	0.6–4.3	2.3	NA
Monocytes (%)	Male	6.0–11.4	8.6	NA	-	NA	NA	-	6.8–21	10.0	5.3–6.4	6.0	4.3–15.2	9.7	NA
	Female	6.0–11.4	8.4	NA	-	NA	NA	-	6.1–26.3	11.0	6.5–7.5	7.0	4.6–17.2	8.8	NA
Monocytes (10^9^/L)	Male	0.2–0.8	0.4	0.1–0.5	0.3	NA	NA	-	NA	-	NA	-	0.2–1.0	0.5	NA
	Female	0.3–0.9	0.5	0.1–0.7	0.3	NA	NA	-	NA	-	NA	-	0.2–1.0	0.5	NA
Platelets (10^9^/L)	Combined	142–354	241	NA	-	126–438	NA	-	NA	-	NA	-	NA	-	150–350
	Male	140–346	231	133–339	211	NA	124–378	225	106–352	218	206.8–227	213	86–348	186	NA
	Female	148–367	253	143–369	243	NA	117–382	228	120–379	227	229–251	236	111–416	214	NA

NA, not applicable; RBC, red blood cell; HGB, haemoglobin; MCV, mean cell volume; MCH, mean cell haemoglobin; MCHC, mean cell haemoglobin concentration; RDW, red cell distribution width; WBC, white blood cell; PI, percentile interval.

The median total WBC for men was lower than that for women, and the difference was statistically significant. This may be attributed to the significant difference in the immune system of men and women, associated with the presence of sex hormone receptors on the immune cells. These make women generally more prone to autoimmunity, resulting in lower rates of infection and chronic inflammatory disease.^45,46^ Our findings are in concordance with those reported by Oloune et al. in Yaoundé, Cameroon,^6^ Bakrim et al. in Morocco,^8^ Tekkeşin et al. in Turkey^14^ and Mine et al. in Botswana.^47^

The significantly higher median platelet count in women compared to men is suggestive of variations in hormone type and concentrations in the different genders as well as the effect of erythropoietin released in response to menstrual blood loss and cross-stimulation of megakaryopoiesis.^10,36^ Our findings are consistent with other studies in Africa: Addai-Mensah et al. in Ghana,^7^ Bakrim et al. in Morocco,^8^ Mulu et al. in Ethiopia,^13^ Miri-Dashe et al. in Nigeria,^36^ Dosoo et al. in Ghana^10^ and Kibaya et al. in Kenya.^17^ However, platelet counts in this study were relatively higher than those of other African countries in contrast to higher counts reported in the United States (Table 5).^5^ This could be attributed to genetic factors, compounded by the increased consumption of platelets by *Plasmodium* spp. in malaria-endemic areas.^48,49^

### Limitations

A limitation for this study was that we could not screen for malaria, helminthes or all types of abnormal haemoglobin (except for the AS and SS sickle cell genotypes), and our complete blood analyser could not differentiate the granulocytes into neutrophils, basophils and eosinophils. Also, subclinical conditions which could affect blood parameters were not discernable during sample collection. Furthermore, ethnic and cultural differences that may influence diet and nutritional practices could have affected the outcome of our haematological intervals. We could not control for potential selection bias for some people who visited Bamenda and donated blood.

### Recommendations

We recommend that locally generated haematological values should be used as reference intervals in our locality and that each region in Cameroon should determine their haematological reference intervals as recommended by the Clinical Laboratory Standards Institute.^21^

### Conclusion

The haematological reference intervals established in this study are comparable to those obtained in Yaoundé, Cameroon and other studies within and outside of Africa. Any differences in values may be due to differences in latitudes of the localities, race and diet. We propose that the present established haematological reference intervals in this study should be used for clinical management of patients and interpretation of laboratory data for research in Bamenda.
